# SARS-CoV-2 vs. Hepatitis Virus Infection Risk in the Hemodialysis Population: What Should We Expect?

**DOI:** 10.3390/ijerph18115748

**Published:** 2021-05-27

**Authors:** Luis D’Marco, María Jesús Puchades, Miguel Ángel Serra, Lorena Gandía, Sergio Romero-Alcaide, Elena Giménez-Civera, Pablo Molina, Nayara Panizo, Javier Reque, José Luis Gorriz

**Affiliations:** 1 Nephrology Department, Hospital Clínico Universitario (INCLIVA), 46010 Valencia, Spain; chuspuchades@gmail.com (M.J.P.); lorenagandiaincliva@gmail.com (L.G.); sergioincliva@gmail.com (S.R.-A.); elenagcivera@gmail.com (E.G.-C.); nayapanizo@gmail.com (N.P.); 2 School of Medicine, Universidad de Valencia, 46010 Valencia, Spain; miguel.a.serra@uv.es (M.Á.S.); molina_pab@gva.es (P.M.); 3 Digestive Medicine Department, Hospital Clínico Universitario de Valencia, 46010 València, Spain; 4 Nephrology Department, Hospital Dr. Peset, FISABIO, 46017 València, Spain; 5 Nephrology Department, Hospital de Castellón, 12004 Castellón, Spain; javier.reque@hotmail.com

**Keywords:** chronic kidney disease, hepatitis, SARS-CoV-2, COVID19, dialysis

## Abstract

Since the dramatic rise of the coronavirus infection disease 2019 (COVID-19) pandemic, patients receiving dialysis have emerged as especially susceptible to this infection because of their impaired immunologic state, chronic inflammation and the high incidence of comorbidities. Although several strategies have thus been implemented to minimize the risk of transmission and acquisition in this population worldwide, the reported severe acute respiratory syndrome coronavirus 2 (SARS-CoV-2) seroprevalence varies across studies but is higher than in the general population. On the contrary, the screening for hepatitis viruses (HBV and HCV) has seen significant improvements in recent years, with vaccination in the case of HBV and effective viral infection treatment for HCV. In this sense, a universal SARS-CoV-2 screening and contact precaution appear to be effective in preventing further transmission. Finally, regarding the progress, an international consensus with updated protocols that prioritize between old and new indicators would seem a reasonable tool to address these unexpended changes for the nephrology community.

## 1. Introduction

Since the onset of the severe acute respiratory syndrome coronavirus 2 (SARS-CoV-2) pandemic, early findings have identified a variety of populations at higher risk. Among these, chronic kidney disease (CKD) patients are in the eye of the storm because of their high incidence of comorbidities such as diabetes or hypertension, even more recurrent in those patients undergoing renal replacement therapies (RRT) [1].

A gold standard in nephrology departments and dialysis units is serology screening for hepatitis B and C virus (HBV/HCV) before starting CKD patients on chronic RRT, to be carried out yearly given the high risk of HBV or HCV infection involved in dialysis procedures. Nonetheless, the current situation is evolving more rapidly than expected. Although several protocols and RRT techniques are safer than in previous decades, and the risk of HBV/HCV infection is very low, the rapid rise of the SARS-CoV-2 pandemic has increased pressure on many care units managing chronic patients such as those on dialysis (Table 1).

Coronavirus infection disease 2019 (COVID-19) has been reported in patients on RRT, and several strategies have thus been implemented to minimize the risk of transmission and acquisition in this population worldwide. Reported SARS-CoV-2 seroprevalence varies across studies but is approximately two to three-fold higher than in the general population [2]. It should be noted that not all patients on chronic dialysis treatments, either hemodialysis (HD) or peritoneal dialysis (PD), are screened [3], meaning that exact COVID-19 rates remain unknown. In addition, CKD patients are known to have impaired immune status, hence development of the humoral response to SARS-CoV-2 has not been previously reported.

Here, we explore the need for new changes and protocols to improve patient care in response to current and future threats, especially in high-risk populations such as patients receiving dialysis, a subset displaying a growing trend towards older age and a greater number of comorbidities.

## 2. Hepatitis Virus in the Dialysis Population

The link between dialysis units and HBV and HCV is well known [4], yet recent years have seen significant improvements, brought about mainly by the advent of highly reliable diagnosis of these infections in renal affected patients receiving dialysis, vaccination in the case of HBV, and effective viral infection treatment, achieving partial response in HBV and complete and sustained response in HCV patients. These developments have allowed a major shift in this interrelationship. It is established that to eradicate a chronic viral infection or reduce it to a residual disease in a particular patient group, three steps are required: early and reliable detection of viral infection, effective treatment of infected patients within the group, and finally, prophylactic vaccination of non-infected patients.

Regarding HCV, for multiple reasons, a high prevalence (13.5%) of this infection has previously been observed in HD units [5], with reports oscillating between 2.6% and 22.9%; however, these high rates have been decreasing during recent years and the current 0.84% is comparable with or even lower than in the general population [4]. This substantial difference is owing to the practices outlined above. First, HCV infection screening is compulsory in all patients, always including antibody and HCV RNA detection [6]. This measure completely eliminates inadvertent nosocomial HCV transmission in patients undergoing HD. The second factor is the availability since 2015 of a HCV treatment effective in more than 95% of cases, administered over 8 or 12 weeks without any negative effect on residual renal function or on other organs [6,7]. This has reduced the probability of infection in dialysis populations during or outside the sessions to a similar level as in patients undergoing medical procedures in other specialties. Considering also that as an RNA virus, HCV reactivation is impossible despite immunosuppressive treatment, HCV screening every six months is of questionable benefit, except in patients where reinfection is an eventuality due to practices outside the HD unit such as intravenous drug use or risky sexual practices in certain subgroups [6]. Therefore, patients with past HCV infection who are HCV RNA-negative do not currently require isolating in the HD unit. It is worth underlining that the presence of antibodies against HCV without HCV RNA is not indicative of active infection; on the contrary, it has shown a definitive cure. In 2021, while HCV infection has not been completely eradicated due to the lack of a vaccine, these measures have reduced it to a residual and perfectly curable disease.

Turning to HBV, the occurrence of this infection in HD units has been established for many years; in the United States, nonetheless, HBV incidence reported in units decreased from 6.2% in 1974 to 1% in 2019 [4,8]. Since then, the situation has improved significantly and a similar prevalence has been observed in European HD units, with no reconversions aside from occasional episodes [9].

Unlike HCV, HBV is a DNA virus, requiring a slightly different prophylaxis and control strategy, although it shares with HCV reliable detection, with serological marker and HBV-DNA testing to rule out occult infection: an exceptional occurrence which, in any case, is not infectious if serum DNA-HBV is undetectable [10,11]. The predominant differential factor of HBV is the availability of a vaccine for patients without current HBV infection (HBsAg and DNA-HBV-negative) or evidence of past infection (Anti-HBc and/or anti-HBs-positive). The best prophylaxis method is the vaccination of patients without signs of HBV infection. Initially, vaccines showed low seroconversion in these patients, but new options with adjuvants and pre-S1 and Pre-S2 proteins achieve a high rate of anti-HBs antibody development, and once achieved, protection from HBV infection is guaranteed [7,8].

In HBV detection, the treatment of chronic infection is feasible, but achieves only a partial response, i.e., HBsAg presence remains, but HBV-DNA (the infecting element) is undetectable, which prevents the patient from transmitting the infection. At present, it is possible to treat chronic HBV infection with DNA-polymerase inhibitor drugs, which maintain control over replicative activity. This has been possible for over 10 years with the use of entecavir or tenofovir, which have shown limited dose adjustment and, in the latter case, possible effects on phosphate and calcium metabolism, although with a low likelihood of resistance development. Currently, tenofovir alafenamide fumarate (TAF) can be administered after hemodialysis without dose adjustment, does not alter renal function or affect phosphate or calcium metabolism, and should be continued over the long term [12,13].

The monitoring of HCV-infected and HCV-cured patients is presently based around the fact that reinfection is exceptional if intravenous drug use and risky sexual practices are avoided, and that specific isolation is not required within the HD unit; it should not be performed in patients without previous HBV infection, vaccinated or cured of HBV infection. Isolation should be reserved for HBsAg-positive patients with or without spontaneous presence of HBV DNA, or on antiviral treatment, and should be evaluated periodically (every six months). Finally, HBV infection can be prevented keeping the current recommendations for prevention and control of the infection in hemodialysis units, with vaccination, and by avoiding sexual contact with infected people and intravenous drug use [14]. The use of disposable material should be prioritized to the utmost, given that the vast majority of outbreaks are related to the use of multipurpose vials and blood puncture with reused material or gloves [8,15].

## 3. SARS-CoV-2 in Dialysis Population

Since the dramatic rise of the COVID-19 pandemic, patients receiving maintenance HD have emerged as especially susceptible to this infection, as the need for periodic attendance to dialysis centers for life support excludes them from social distancing and isolation measures. Additionally, dialysis centers are high-risk settings with shared rooms which increase the risk of contact among infected people. In this regard, of 7154 patients undergoing HD in 65 centers in Wuhan, China, 154 had a laboratory-confirmed test for SARS-CoV-2 (2.15%) [16]. Of note, in May 2020 ∼2.9% of all patients on dialysis in Europe were SARS-CoV-2-positive cases (country range, 1.0–3.7%) [17]. In Spain, 36 (12.75%) of 282 patients followed in two reference hemodialysis units were hospitalized for COVID-19 [18]. In a large-size London HD center, of 1530 patients receiving HD, 300 (19.6%) developed COVID-19 [19]. The Open SAFELY platform, which gathers data from 17 million people affected by COVID-19 worldwide, showed that end-stage renal disease, dialysis and kidney transplantation almost quadrupled mortality risk compared to the general population and increased mortality by 50–100% in comparison with other reported risk factors for COVID-19 severity such as obesity, hypertension and diabetes [20,21,22]. Notably, healthcare teams in HD units have also shown increased risk, as illustrated by data from a Canadian urban HD center, in which 93 (12%) staff members had a positive RT-PCR test for SARS-CoV-2 [20].

Dialysis patients have shown chronic immune dysfunction [16] and inflammation, and are more exposed to COVID-19-related complications due to their older age and comorbidities [1,23], resulting in higher reported mortality rates (approx. ≤20%) [17]. Nevertheless, the severity of the clinical course of this novel disease is largely determined by the cytokine storm and uncontrolled acute rather than chronic inflammatory state. This allows the hypothesis that the clearance of certain molecules such as cytokines from the plasma of COVID-19 patients could exert an impact on outcomes and mortality. Indeed, a recent study including COVID-19 patients on maintenance HD who were dialyzed with polymethyl methacrylate (PMMA) filters found the highest mortality in the patient subset unable to reduce IL-6 levels despite the adsorption attributable to this kind of filter [24]. No studies have yet compared the mortality of HD patients based on the type of dialysis received (online hemodiafiltration vs. high-flow HD). Moreover, evidence remains scarce regarding an effective treatment for SARS-CoV-2.

Several vaccines are already available, with SARS-CoV-2 spike protein as the main target. They work by different mechanisms such as mRNA-based, vector-based or protein-based vaccines [25]. Unfortunately, CKD patients were largely excluded from Phase 3 trials of these vaccines, which makes it difficult to define a specific protocol in this population. However, some countries have included CKD patients on the priority list for vaccination, since side effects are mild and similar to those reported in other high-risk populations.

It has been postulated that the vaccine could be less effective in CKD, dialysis and kidney transplanted populations due to a previously reported uremic-related immune defect and/or immunosuppressive medication. Nevertheless, these patients may particularly benefit from the vaccine given their increased risk for severe or fatal COVID-19. In this regard, one study reported a weak anti-SARS-CoV-2 antibody response for a mRNA COVID-19 vaccine in kidney transplant recipients after the first injection [26].

These points remain to be established: 1. If the response to the vaccine against SARS-CoV-2 is similar in all stages of CKD and modalities of renal replacement therapy; 2. Determining a cut-off point for protective antibodies that indicate the need for a new dose of vaccination, as is currently the case of HBV, or if additional cellular immunity screening are required for establishing the need of vaccination; 3. The need for periodic vaccination against SARS-CoV-2, and; 4. If in patients with CKD this strategy is similar to the general population.

Further studies are required to answer these questions and warranted to explore these interesting findings, which may prevent the high mortality rates observed in patients on dialysis or with immunosuppressive status.

## 4. Conclusions

**Figure 1:**
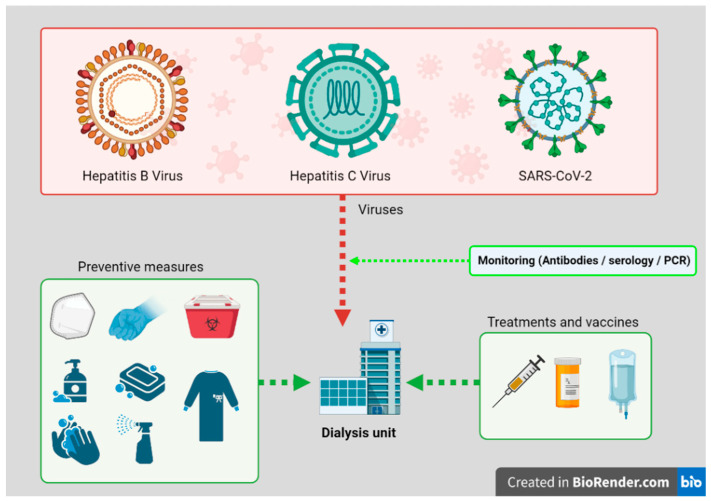
Preventive measures and treatment options against Hepatitis and SARS-CoV-2 viruses for dialysis units.

In summary, universal SARS-CoV-2 screening and contact precaution in case of outbreaks appear to be effective in preventing further transmission (Figure 1). The burden of immune deficit, chronic inflammation and multiple comorbidities stand out as the main concerns in CKD and dialysis patients, urging close surveillance in all risk groups. Finally, regarding the progress, nephrologists can hope to see during this global threat an international consensus with updated protocols that prioritize between old and new indicators, which would seem a reasonable tool to address these unexpended changes.

## Figures and Tables

**Table 1:** Infection prevention and risk.

	HBV	HCV	SARS-CoV-2
**Infection risk**	Low	Low	High
**Mortality risk**	Low	Low	High
**Routine serology**	Yes	Yes	No/?
**Patient screening**	Yes	Yes	No
**Staff screening**	Yes	Yes	No
**Vaccination**	Yes	---	No/??
**Effective treatments**	++	+++	+-

Effective: Unknow (+-); moderate (++); Very effective (+++).
